# Draft Genome Sequence of Uropathogenic *Escherichia coli* Strain J96

**DOI:** 10.1128/genomeA.00245-12

**Published:** 2013-02-21

**Authors:** Eric A. Klein, Zemer Gitai

**Affiliations:** Department of Molecular Biology, Princeton University, Princeton, New Jersey, USA

## Abstract

*Escherichia coli* J96 (O4:K6) was isolated from a human pyelonephritis patient. Here, we report the draft genome sequence of *E. coli* J96, which contains virulence genes, including adhesion factors, alpha-hemolysins, and cytotoxic necrotizing factor. J96 infects the kidney and bladder, making it an important tool for studying *E. coli* pathogenesis.

## GENOME ANNOUNCEMENT

Uropathogenic *Escherichia coli* (UPEC) strains are the most common bacteria that cause urinary tract infections. UPEC strains differ primarily by the presence of various virulence factors, often encoded on pathogenicity islands (PAIs). *E. coli* strain J96 was isolated from a human pyelonephritis patient (1). Several studies have characterized J96 virulence factors, including adhesins (*pap*, *prs*, *foc*, and *fim*), alpha-hemolysins (*hly*), and the cytotoxic necrotizing factor (*cnf*) (2, 3). In addition to chromosomal pathogenicity factors, J96 carries an ∼115-kb plasmid (pJ96) (4). The variety of virulence genes, together with the ability of J96 to infect bladder and kidney tissues, makes this strain a valuable tool for studying UPEC pathogenesis. A previous work produced a physical-genetic map of the J96 genome (4), as well as the sequences of several of pathogenic factors (5, 6). In this report, the full chromosome and plasmid sequences were subjected to genomic analysis.

Three paired-end libraries were prepared: (i) total DNA (200 bp), (ii) total DNA (500 bp), and (iii) plasmid DNA (200 bp). The genome sequence was determined using shotgun sequencing (read length, 101 bp) on an Illumina HiSeq 2000 (library 1, 116,959,856 reads and 2,300× coverage; library 2, 85,721,146 reads and 1,690× coverage; plasmid, 30,608,372 reads and 26,800× coverage). The reads were quality-filtered and randomly sampled to achieve 100× coverage for the assembly. The plasmid sequence was assembled using Edena v3 (7), resulting in 14 contigs. Plasmid contaminants in the total DNA libraries were removed by aligning reads to the plasmid contigs using Bowtie (8). The chromosomal reads were assembled in two phases. *De novo* assembly was done with Edena v3 (7), Assembly by Short Sequences (ABySS) v1.3.1 (9), and Velvet v1.2 (10). Reads were also aligned to UPEC strains UTI89 (accession no. NC_007946), CFT073 (accession no. NC_004431) (11), and EC536 (accession no. NC_008253). The *de novo* assemblies and alignment-based contigs were merged using Gap4 (12) and scaffolded with SSAKE-based Scaffolding of Pre-Assembled Contigs after Extension (SSPACE) v2.0 (13), and gaps were PCR-amplified and sequenced by Sanger sequencing. The assembly of chromosomal sequences resulted in 262 contigs, which were annotated via the NCBI Prokaryotic Genome Annotation Pipeline (PGAAP) (14). The J96 chromosome is 5.51 Mb and its G+C content is 51.1%. pJ96 is 112 kb and its G+C content is 50.9%. The chromosome and plasmid contain 5,259 and 140 protein-coding sequences, respectively. The chromosome also contains 28 rRNA and 105 tRNA genes.

Phylogenetic analysis based on average nucleotide index (ANI) values (15) among *E. coli* strains showed that J96 is most similar to other UPEC strains. At least three PAIs were identified: PAI-I (*pap*), PAI-II (*hly, prs*, *cat-1* [chloramphenicol resistance], and *fim*), and PAI-III (*foc*). Additionally, the genome encodes numerous transposases and insertion sequences, highlighting the importance of genomic exchange in creating the J96 pathogenic phenotype.

Alignment of pJ96 to other UPEC plasmids revealed high similarity to pUTI89 (accession no. NC_007941), though pJ96 lacked several hypothetical proteins (P018-020 and P084-085) and contained 6 single nucleotide polymorphisms (SNPs).

### Nucleotide sequence accession numbers. 

*E. coli* strain J96 can be obtained from ATCC (no. 700336). This Whole Genome Shotgun project has been deposited at DDBJ/EMBL/GenBank under the accession no. ALIN00000000. The version described in this paper is the first version, ALIN02000000.
